# Protective effect of curcumin on busulfan-induced renal toxicity in male rats

**DOI:** 10.1080/0886022X.2020.1818580

**Published:** 2020-09-20

**Authors:** Mohammad-Amin Abdollahifar, Vahid Ebrahimi, Abbas Aliaghaei, Amir Raoofi, Amirreza Khosravi, Amirhosein Hasani, Ali Mehdizadeh, Mohammad Asadi

**Affiliations:** a Department of Biology and Anatomical Sciences, School of Medicine, Shahid Beheshti University of Medical Sciences, Tehran, Iran; b Department of Anatomy and Cell Biology, School of Medicine, Mashhad University of Medical Sciences, Mashhad, Iran; c Leishmaniasis Research Center, Department of Anatomy, Sabzevar University of Medical Sciences, Sabzevar, Iran; d Student Research Committee, Department and Faculty of Medical Sciences, Shahid Beheshti University of Medical Sciences, Tehran, Iran

**Keywords:** Busulfan, curcumin, male rats, renal toxicity

## Abstract

**Aim:**

The aim of this study was to evaluate the effects of curcumin in an experimental model of busulfan-induced renal toxicity with emphasis on importance of histological alterations.

**Methods:**

In this study, we utilized 32 adult male Wistar rats (250 ± 10 g). All the animals were divided into four experimental groups randomly: (I) Control; (II) Busulfan (40 mg/kg); (III) Olive oil; and (IV) Curcumin (80 mg/kg/day). Finally, the rats were euthanized and kidney tissues were taken for histopathology experiments, serum BUN, and creatinine level, reactive oxygen species (ROS) production and glutathione disulfide (GSH) activity.

**Results:**

Our result showed that the reduction in body weight and kidney weight in busulfan groups in comparison with the control and curcumin groups. The result in this study also showed that the reduction in BUN, creatinine, and ROS production in curcumin groups in comparison with the busulfan group together with an increasing of GSH activity compared to busulfan induced rats.

**Conclusion:**

Our results of this study indicated that that the reduction in body weight, kidney weight, total volume of kidney, total length of nephron tubules, and numerical density of glomeruli and nephron tubules in busulfan groups in comparison with the control and curcumin groups However, curcumin can be an alternative choice for therapeutically and research purposes in the disturbing kidney after treatment with busulfan.

## Introduction

Busulfan is a chemotherapy drug and it is a cell cycle nonspecific alkylating antineoplastic agent, which is a chemical designation is 1,4-butanediol dimethanesulfonate and it is in the class of alkyl sulfonates [1]. The chemical structure of busulfan contains two easily displaced methane sulfonate groups on opposite ends of a butane chain. Hydrolysis of these groups produces highly reactive, positively charged carbonium ions that alkylate and damage DNA molecules. The busulfan reacts with guanine molecules and cysteine molecules on histone proteins through a nucleophilic substitution reaction, forming DNA intrastrand or interstrand cross-links [2]. Busulfan by alkylating activity can produce alterations of cell replication, gene transcription, and DNA damage repair [1].

The impact of chemotherapy on improving and prolonging the life of patients with kinds of cancers is undeniable, but it often involves severe side effects including cognitive problems, early menopause, heart diseases, bone and joint disorders, secondary cancers, disrupt the blood–testis barrier, and renal diseases [3–5].

Nephrotoxicity caused by chemotherapeutic agents makes a significant complication limiting the efficacy of the treatment [6]. A variety of disorders associated with renal function can results from some chemotherapeutic agents such as busulfan, including inflamed kidney tissue, papillary necrosis, urothelial alterations, hemorrhagic cystitis, acute tubular necrosis, and also infarction [6,7]. The clinical observations indicated that cancer patients treated with busulfan/Cyclophosphamide (Bu/Cy) can potentially suffer from ovarian failure, infertility, and renal dysfunction. It means that the use of Bu/Cy compound affects the ovarian function and also it has been made clear that Bu/Cy combination has side effects on the several organs such as spleen, lungs, and kidneys. Although, it does not seem to have much impacts on the heart, liver, stomach, and pancreas [8,9]. In previous studies, histological assessments of animal model of ovarian failure induced by Bu/Cy demonstrated impairments in ovarian tissue. A sharp edema in the cortical and medullary layers of kidneys, shrinkage of the glomeruli, and persistent edema in the tubular structures were also reported. Due to increasing use and on the other hand, the side effects of chemotherapeutic drugs, somewhat safer natural products provide an appropriate and rich origin for exploring adjuvant factors that hinder and decrease the side effects of chemotherapy. In this regard, curcumin can be suggested [10,11]. Curcumin is a functional polyphenolic compound from the plant Curcuma longa which has the usage as a natural remedy for a long time in various Asian regions owing to its curative effects on disorders such as diabetes, liver and kidney diseases, rheumatoid diseases, infectious diseases, and cancer [12–14]. Several studies have shown that curcumin has potent anti-oxidative, anti-microbial, anti-inflammatory, and anticancer operations. Furthermore, current studies have shown that curcumin is capable to hinder carcinogenesis in sensitive cells after chemotherapy and protects normal cells from the damages caused by chemotherapy [12,13]. According to antioxidative and anti-inflammatory properties of curcumin, a protective effect against kidney damages and renal toxicity following chemotherapeutic drugs administration has been reported [15,16]. Furthermore, it has been revealed that curcumin is able to show protecting effects against ischemic renal impairment by upregulation of antioxidant gene expression, mainly Mn-superoxide dismutase, the antioxidant enzyme in the mitochondrial matrix [15,16]. For the reason that many people are suffering worldwide from side effects of chemotherapy drugs, the aim of this study was to estimate the effects of curcumin in an experimental model of busulfan-induced renal toxicity with emphasis on importance of histological alterations.

## Methods and materials

### Animal models

In this study, 32 adult male Wistar rats (250 ± 10 g) were collected from the laboratory animal center of Pasture Institute and were approved by the the Medical Ethics Committee at Shahid Beheshti University of Medical Sciences, Tehran, Iran (IR.SBMU.MSP.REC 1399.027). The rats were divided into four experimental groups: Group I (Control group): The animals of this group were intact. Group II (Busulfan group): The animals of this group were received a three-dose of busulfan (40 mg/kg) in 0 day, 7 days and 21 days intraperitoneally (IP). Group III (Olive oil): The rats of this group were received a three dose of busulfan 40 mg/kg IP + daily receiving olive oil by gastric gavage for 30 days after the last injection of busulfan. Group IV (Curcumin): The animals of this group were received a three dose of busulfan 40 mg/kg IP) + curcumin 80 mg/kg/day, which was dissolved in olive oil by gastric gavage for 30 days after the last injection of busulfan. Each group comprised eight rats that were kept in under standard situation, at room temperature (22–24 °C), and with a 12:12-h light-dark schedule; the animals also had optional availability to water and food. The rats’ body weights were monitored at first of study and the end of study. The kidney weights were also measured at the end of study.

### Blood collection

At the end of the experiment, rats were anesthetized by ketamine (100 mg/kg) and xylazine (5 mg/kg) and then blood samples were obtained from the left ventricle of heart. The blood samples were allowed to coagulate and then centrifuged at 3000 rpm for 10 min. The Serum were separated for measurement of blood urea nitrogen (BUN) and serum creatinine.

### Reactive oxygen species in testicular tissue

After isolation testis cells with trypsin EDTA samples with PBS for 5 min at 4 °C was centrifuged at 1200rpm. Then the DCFDA was added to the sample at a concentration of 20 µM in a 100 µl aliquot and stored in a 37 °C incubator for 45 min in the dark. Finally, the sample was examined by a flow cytometer with a wavelength of 495 nm.

### Glutathione disulfide content assessments

GPX assay kit (Zelbio GmbH) was used to determine GPX in testis tissue samples with 5 U/ml sensitivity (5KU/L). In this essay, GPX activity unit was considered as the amount of the sample that will catalyze decomposition of 1µmole of GSH to GSSG in one minute. Aliquots of the testicular cells suspension (0.5 mL) that were previously stained with OPA and NEM probe (5 μM) were separated from the incubation medium by 1 min centrifugation at 1000 rpm. The cell pellet was then suspended in 2 mL of fresh incubation medium. This washing process was carried out twice to remove the fluorescent dye from the media. Each sample was measured in quarts cuvettes using a Shimadzu RF5000U fluorescence spectrophotometer set for at 495 nm excitation and 530 nm emission wavelengths.

### Tissue preparation

At the end of the experiment, the animals were euthanized and their kidneys were collected and fixed in paraformaldehyde 4%. Then the kidneys were fixed in paraffin blocks and cut into 5 and 25 μm thick segments with a usage of microtome. For the microscopic definitive analysis of each group, slides were stained with hematoxylin and eosin (H&E).

### Estimating the volume of kidney and interstitial tissue

By using a microscope connected to a camera, the live image of each kidney section was evaluated. By systematic uniform random sampling (SURS), 8–10 sections were obtained and analyzed for each kidney. Using the stereological software designed at Stereology center (Department of Biology and Anatomical Sciences, School of Medicine, Shahid Beheshti University of Medical Sciences, Tehran, Iran). Total volumes of the kidney and interstitial tissue were assessed using the Cavalieri method. The volume of the kidney and interstitial tissue was estimated using the following formula [17,18]:

$$V_{\mathrm{total}}=\sum P\times t\times \frac{a}{p},$$

where ΣP is the total points hitting the kidney sections, a/p is the area correlated with each point, and t is the distance between the sampled sections.

### Estimating the length of nephron tubules

The length density of the nephron tubules was estimated by superimposed an unbiased counting frame randomly on the live image of each kidney section. The length density of tubules using the formula [17,19]:

$$L_{v}=\frac{2\times \sum Q}{\sum P\times \frac{a}{f}},$$

where ΣQ is the total number of the nephron tubules, a/f is the area per frame, and ΣP is the total points superimposed on the kidney tissue. We estimated the total length of the tubules by multiplying the length density (Lv) by the total volume of the kidney. The total length of the tubule ‘L’ was calculated by multiplying the length density (LV) by the total volume of the tubules.

$$L=L_{v}\times V_{\mathrm{total}}.$$

### Estimating the number of glomeruli and nephron tubules

The total number of glomeruli and nephron tubules was determined using the optical disector method. An unbiased counting frame was superimposed on the live image of each kidney section. To measure the z-axis traveling, we used a microcator was attached to the stage of the microscope. The height of the disector was defined as the section thickness excluding the upper and lower 20% was considered as the guard zone. Using the following equation, the number density (Nv) of different cell types was estimated [17,20]:

$$Nv=\left[\frac{\sum Q^{-}}{\sum P\times \frac{a}{f}\times h}\times \frac{t}{BA}\right],$$

where ΣQ is the total number of the counted glomeruli and tubules, h is the tissue thickness considered for counting, a/f is the area per frame, and ΣP is the total number of the counting frames in all fields. Also, h is the height of the dissector, t is the real section thickness measured using the microcator when the Q − is counted, and BA is the tissue section thickness. The result of the equation was then multiplied by the total volume of the kidney to acquire the total number of glomeruli and nephron tubules.

$$N_{\mathrm{total}}=N_{v}\times V_{\mathrm{total}}.$$

### Statistical analysis

Data were analyzed using One-Way ANOVA and the Kruskall–Wallis test. Results were rendered as average ± SD. The significance of comparisons was denoted by *p* ≤ .05.

## Results

### Body weight and kidney weight

**Figure 1.**
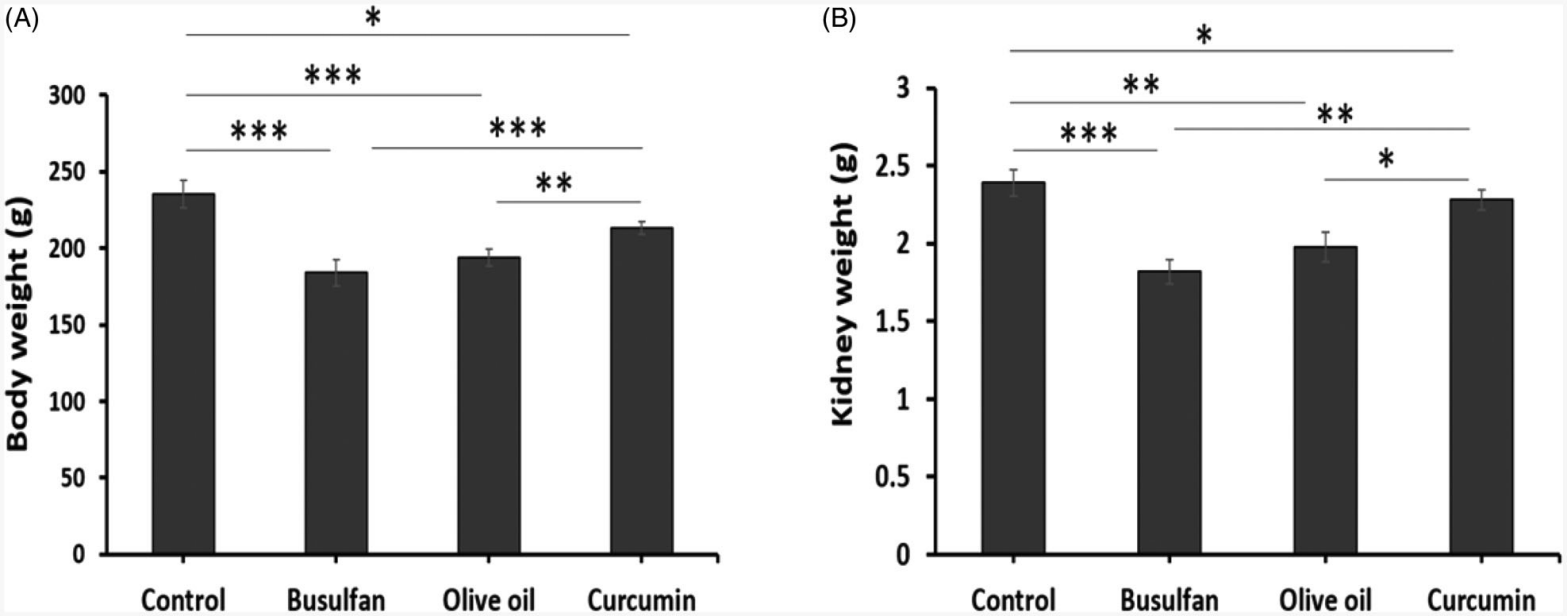
The effects of curcumin on the busulfan-induced impairment in body weight and kidney weight. Mean ± SD of the body weight of rats and the weight of kidney in study groups (*p < .05, **p < .01, and ***p < .001). In the study groups (six animals per group) as compared by the ANOVA and LSD. ANOVA: analysis of variance; CM: conditioned medium; LSD: least significant difference.

Busulfan treated rats from groups 2, 3, and 4 had significantly decreased body weight and kidney weight at the conclusion of the study (*p* < .05; Figure 1). Our results of this study indicated that the decrease in body weight and kidney weight in busulan groups compared to the control and curcumin groups (Figure 1).

### Assessment of biochemical parameters

**Figure 2.**
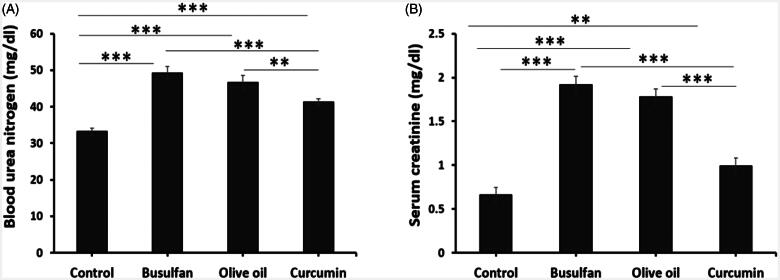
The effects of curcumin on the busulfan-induced impairment in BUN and serum creatinine. Mean ± SD of the BUN and serum creatinine in study groups (*p < .05, **p < .01, and ***p < .001). In the study groups (six animals per group) as compared by the ANOVA and LSD. ANOVA: analysis of variance; CM: conditioned medium; LSD: least significant difference.

The data represented in Figure 2, revealed that administration of busulfan at a dose of 40 mg/Kg significantly increase BUN and creatinine when compared to control and curcumin groups (*p* < .05). Whereas, curcumin was none notably increased BUN and creatinine in comparison with the control groups (*p* < .05). Interestingly, treatment of rats with curcumin significantly decreased BUN and creatinine (Figure 2).

### Effect of scrotal hyperthermia on reactive oxygen species (ROS) production

**Figure 3.**
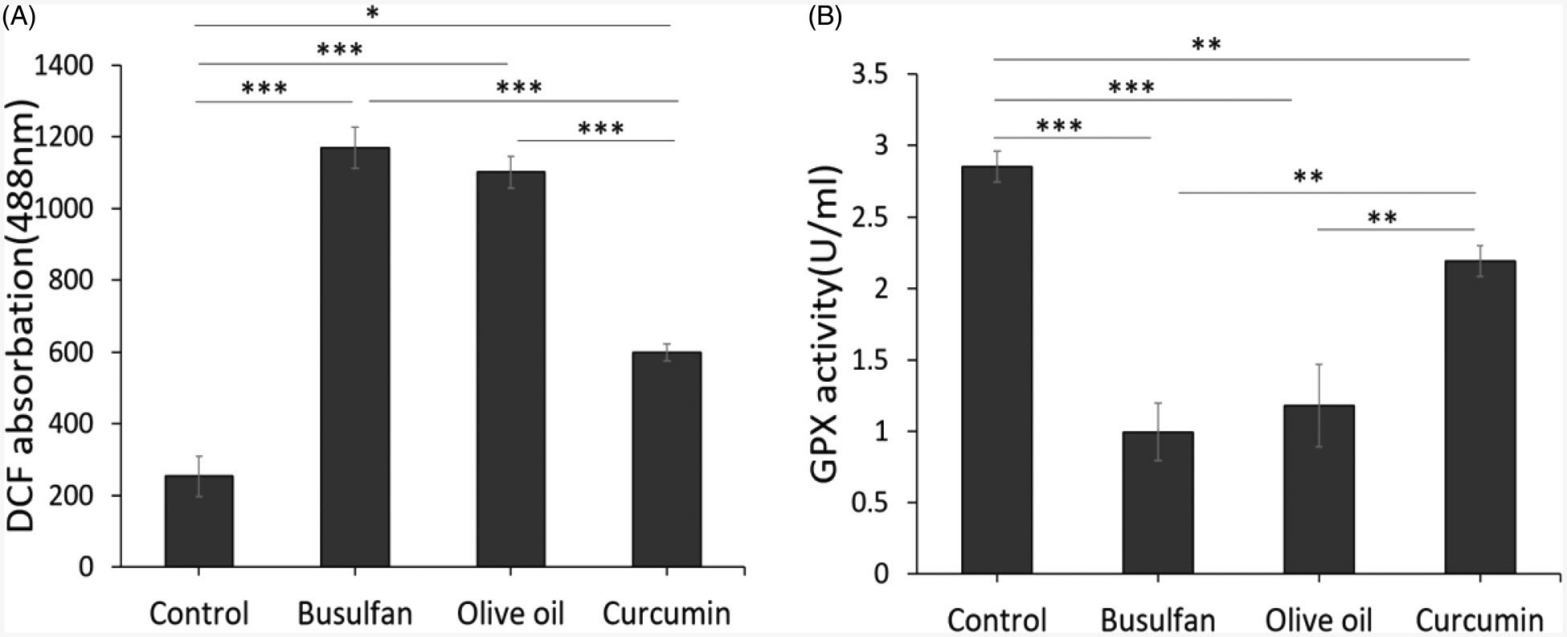
The effect of curcumin on ROS production and level of GSH in study groups. (A and B) Mean ± SD of the ROS production and GPX activity of kidney in the study groups (*p < .05, **p < .01, and ***p < .001).

The effect of busulfan on the formation of ROS was assayed, using flow cytometry. The significant peak shifting shows that, ROS production in the kidney tissue exposed to busulfan compared to control and curcumin groups significantly increased (*p* < .001, *p* < .001), respectively (Figure 3(A)). The results also revealed that the reduction in the ROS production in curcumin groups in comparison to the busulfan and olive oil (*p* < .001, *p* < .001), respectively (Figure 3(A)). Also, there was significant different between Control group with curcumin group (*p* < .05; Figure 3(A)). Our results of ROS assay showed remarkable improve rate following curcumin treatment.

### Thiols metabolism

We assessed concentration of glutathione (GSH) in different groups. As Figure 3(B) shows, concentration of GSH in busulfan group significantly reduced in comparison to control and curcumin groups (*p* < .001, *p* < .01; Figure 3(B)). The results of this study also showed that the GSH significantly decreased in olive oil group in comparison with control and curcumin groups (*p* < .001, *p* < .01; Figure 3(B)). Nonetheless, there is a significant difference between control group comparison to the curcumin groups (*p* < .01; Figure 3(B)). Antioxidant assay of this study showed remarkable improvement rate following curcumin therapy.

### Total volume of kidney and interstitial tissue

**Figure 4.**
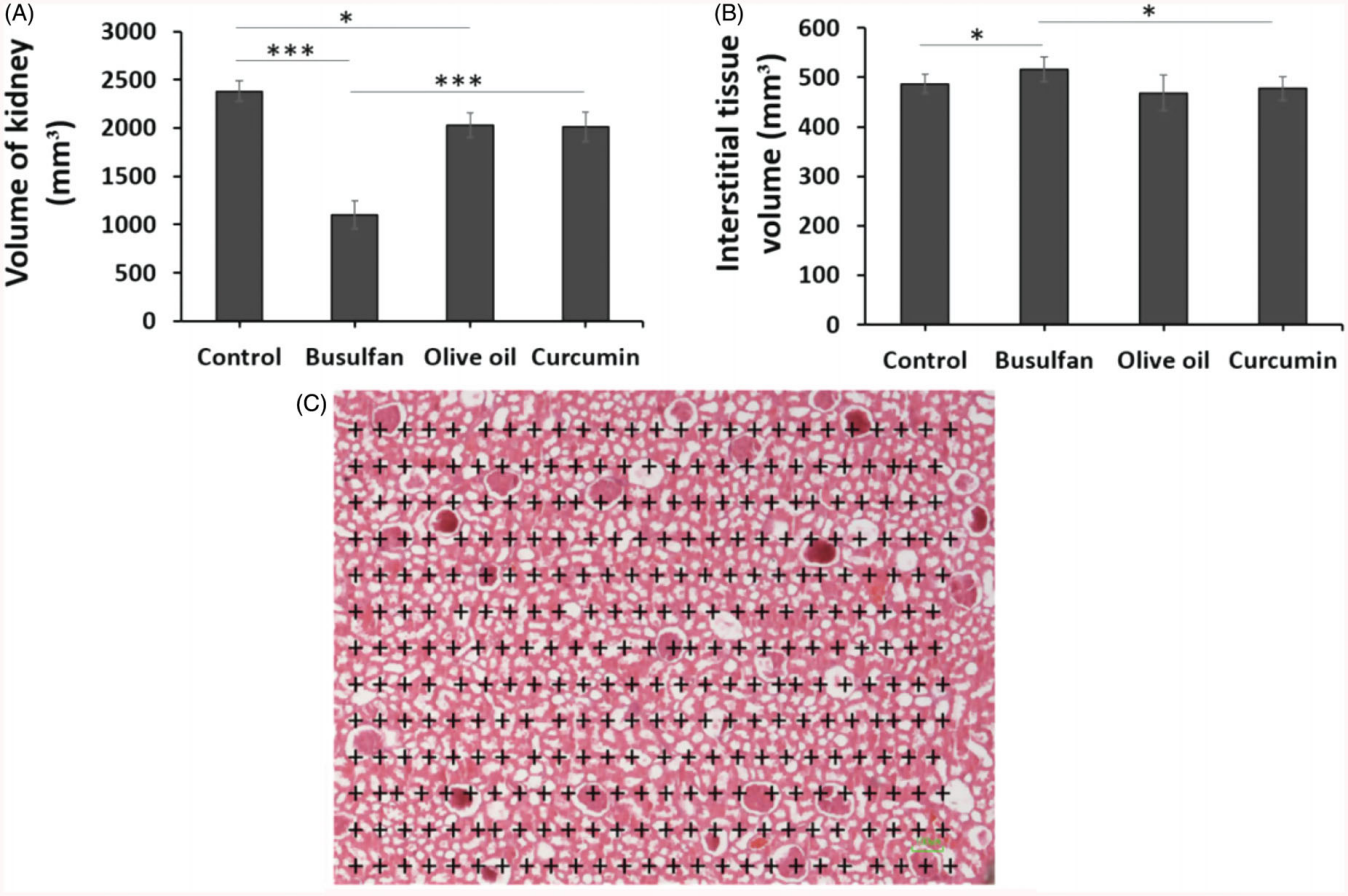
(A and B) The effects of curcumin on the busulfan-induced impairment in total volume of kidney and interstitial tissue. Mean ± SD of the total volume of kidney and interstitial tissue in study groups. (*p < .05, **p < .01, and ***p < .001). In the study groups (six animals per group) as compared by the ANOVA and LSD. (C) A schematic figure showing the protocol of study for Cavalieri method. ANOVA, analysis of variance; CM, conditioned medium; LSD, least significant difference.

The total volume of kidney was remarkably reduced in the busulfan groups in comparison with the control and curcumin groups (*p* < .05). Administration of curcumin in the busulfan + curcumin groups ameliorate the reduction of kidney volume in comparison with the busulfan group, but the volume of interstitial tissue increased after the busulfan injection in comparison with the control and curcumin groups (*p* < .05; Figure 4).

### Total length of nephron tubules

**Figure 5.**
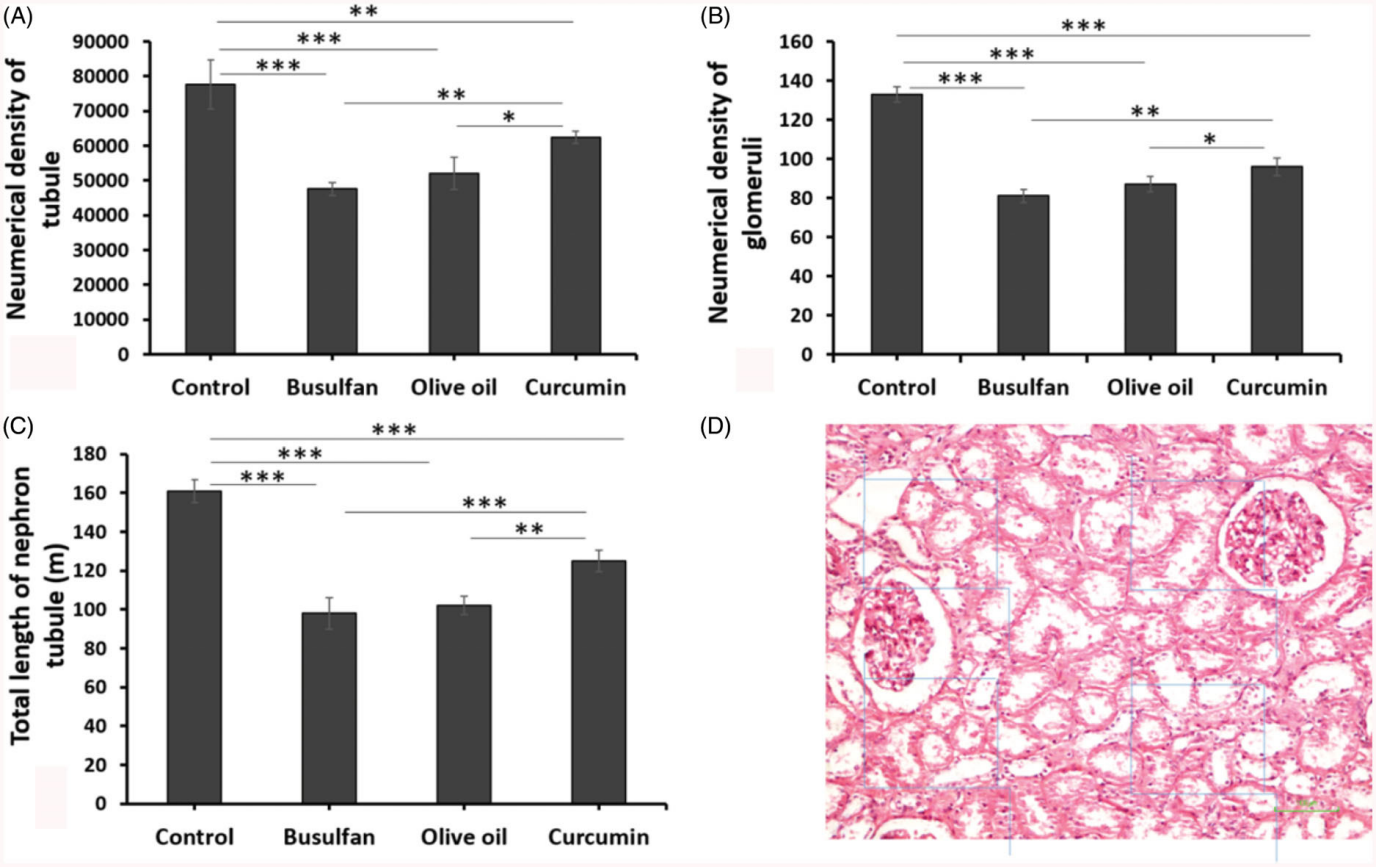
(A–C) The effects of curcumin on the busulfan-induced impairment in the number of glomeruli, nephron tubules, and tubule length. Mean ± SD of the number of glomeruli, nephron tubules, and tubule length in study groups (*p < .05, **p < .01, and ***p < .001). In the study groups (six animals per group) as compared by the ANOVA and LSD. (D) A schematic figure showing the protocol of study for optical disector method. ANOVA: analysis of variance; CM: conditioned medium; LSD: least significant difference.

**Figure 6.**
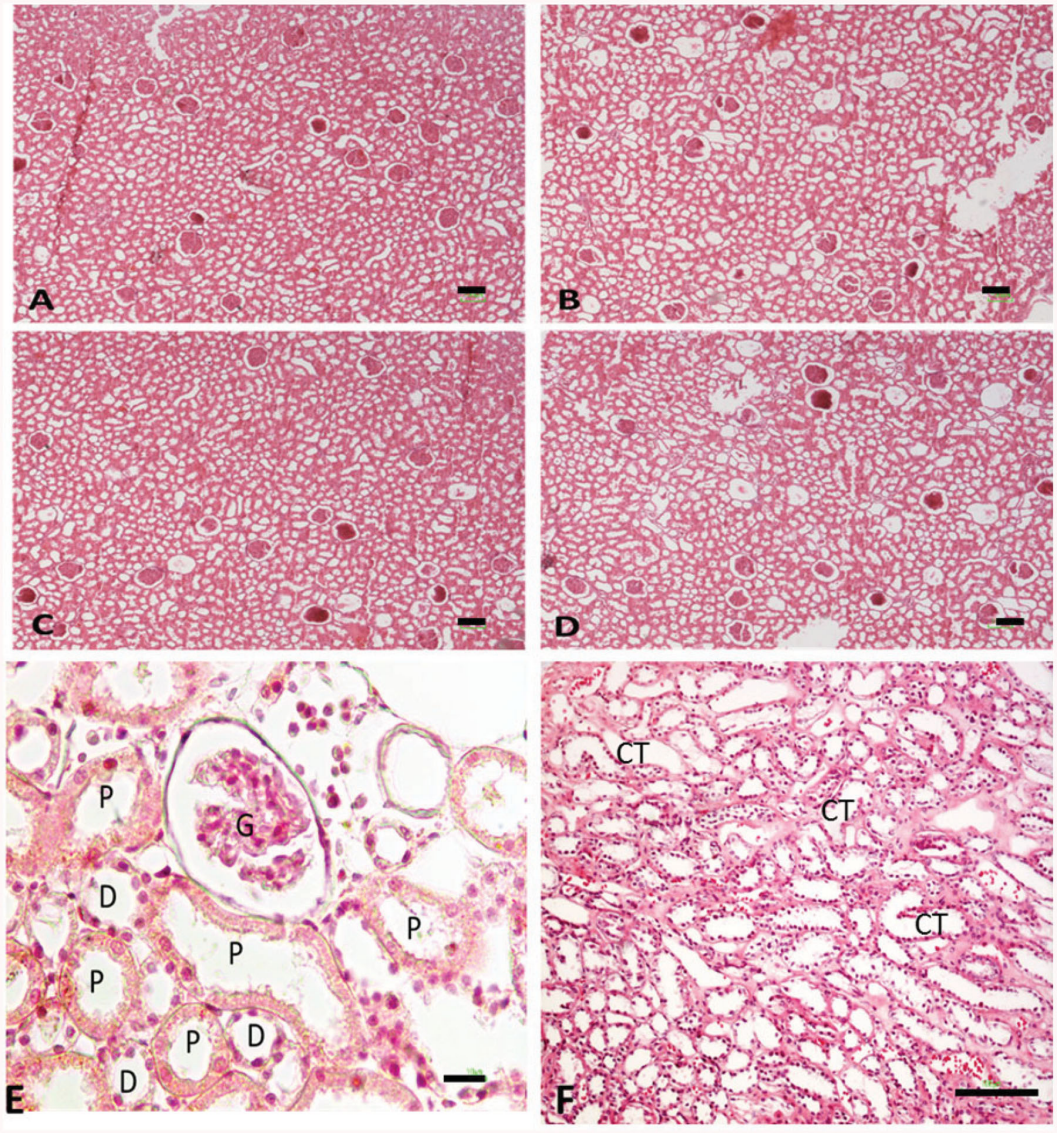
Photomicrograph of the testis stained with H&E (40×). (A–D) Control group, (D–F) Glomeruli (G), proximal convoluted tubule (P), distal convoluted tubule, collecting tubule (CT).

The total length of nephron tubules was notably reduced in the rats treated with busulfan in comparison with the control and curcumin groups (*p* < .05). The total length of nephron tubules increased after the administration of curcumin in comparison with the busulfan group (*p* < .05; Figures 5 and 6).

### Number of glomeruli and tubules

The numerical density of glomeruli and nephron tubules in animals with busulfan treated decreased in comparison with the control and curcumin groups (*p* < .05). Likewise, the reduction of numerical density of glomeruli and nephron tubules averted after the administration of curcumin in comparison with the busulfan group (Figures 5 and 6).

## Discussion

Acute and chronic renal failure are commonly challenged during chemotherapy. Hence, an essential consideration in limiting the use of such drugs. While some approaches exist to prevent nephrotoxicity, no suggested definite treatment is currently presented [15]. Curcumin, an attractive natural herbal component, is recognized to have anti-inflammatory, anti-apoptotic, and antioxidant possessions in different pathologies [20]. In the present study, we explore the effectiveness of curcumin therapy for the potential treatment for tissue damages associated with chemotherapy, particularly nephrotoxicity.

In several researches, it has been stated that curcumin averts and ameliorates the severity of cisplatin-induced nephrotoxicity, through preventing cytokines and chemokines such as TNF-α and IL-1β and also scavenging free radicals [20,21]. Although the major mechanisms of chemotherapeutic agents-induced nephrotoxicity are complex, previous studies have demonstrated the role of several interconnected aspects involved in chemotherapeutic drugs-induced renal damages [22]. These prominence causes include toxic effects on renal cells, DNA and mitochondrial damages, inflammation, oxidative stress, apoptosis, and related pathways [23–25]. In this study, the most important findings are that curcumin treatment ameliorates the following conditions related with busulfan-associated renal toxicity: The first, histological and structural improvement of acute renal damages and the next one, renal dysfunction (kidney weight and biochemical parameters like blood urea nitrogen (BUN) along with serum creatinine).

According to our studies, this seems to be the first report based on stereological evaluations regarding the effects of curcumin on renal cellular structure in busulfan-induced nephrotoxicity. For this purpose, estimating the volume of kidney and its interstitial tissue, estimating the length of nephron tubules, and estimating the number of glomeruli and nephron tubules were done by means of specific stereological techniques. According to our data, it was obtained that the total volume of kidney, the total length of nephron tubules, and the numerical density of glomeruli and nephron tubules were significantly reduced following injection of busulfan in rats. On the other hand, administration of curcumin was capable to preclude these morphological atrophy of kidney in comparison with the busulfan group, but the volume of interstitial tissue increased after the busulfan injection in comparison with the control and curcumin-treated rats. Also in this study we perceived an increase in GSH following the treatment of the mice with curcumin and likewise in this study we observed the reduction in ROS production, in curcumin-treated rats.

Based on a previous probe, it has been demonstrated that following simulation of a mouse model of ovarian failure induced by mixture of Bu/Cy administration as a chemotherapeutic combination, after 4 weeks of treatment, a sharp edema of the cortical and medullar layers was observable in the kidneys, and after 12 weeks’ histological examinations of kidneys confirmed the shrinkage of the glomeruli and persistent edema in tubules. In Yan Jiang *et al.* study less effect of Bu/Cy treatment on kidneys and also edema in the renal tubules after 30 days of treatment was noticeable [8]. Reviewing the works about properties and effects of curcumin on urinary system disclosed the role of curcumin in prevention of renal fibrosis through deactivation of TGF-b1-induced epithelial mesenchymal transition process in tubular cells by affecting the snail-1, different interleukins (ILs), and matrix metalloproteinases (MMPs) [20,26]. According to the previous studies, epithelial mesenchymal transition not only is an essential developmental phase of kidney, but also has a significant role in several adult pathologies, mainly renal cancer and fibrosis [26].

Histopathological findings and evaluating renal function parameters in Yeter et al. study revealed that following exposure to a single dose of cisplatin, a chemotherapeutic drug, tubular necrosis, luminal dilatation, and inflammatory cell infiltration in the rat kidneys were detected, particularly in the corticomedullary area [22]. Subsequently, the curcumin treatment significantly decreased the harshness of damages. Furthermore, serum urea and creatinine values of the cisplatin-induced group were significantly increased [20]. And this increase was significantly reduced after curcumin administration. It has been made clear that one of the potential mechanisms associated with chemotherapeutic drugs such as cisplatin and busulfan nephrotoxicity, is the creation of reactive oxygen species (ROS), also diminution of function of the antioxidant system, and finally, accumulation of lipid peroxidation in the kidneys [27–29].

Further experimental outcomes from Zhou *et al.* exhibited that dose-dependently treatment with chemotherapeutic agent mitomycin (MMC) amplified the levels of creatinine (Cr) and blood urea nitrogen (BUN) in mice, signifying that MMC provoked complicated injuries to the kidneys. After administration of curcumin, the levels of Cr and BUN were notably declined and became like the healthy samples. On the other hand, the impairments were less apparent in the experimental group of combination of curcumin and cisplatin treatment, obviously demonstrating a prospective activity of curcumin in preventing and even cure of renal injuries [30]. Their results more confirm that curcumin can be considered as a potential natural drug and ameliorates chemotherapy-associated nephrotoxicity.

## Conclusions

Therefore, it can be concluded that the curcumin showed protective effect on histopathologic changes and BUN and creatinine levels and also ROS production and GSH activity of rat kidney exposed to busulfan. However, curcumin can be an alternative choice for therapeutically and research purposes in the disturbing kidney after treatment with busulfan.
